# The Joint Effects of Lifestyle Factors and Comorbidities on the Risk of Colorectal Cancer: A Large Chinese Retrospective Case-Control Study

**DOI:** 10.1371/journal.pone.0143696

**Published:** 2015-12-28

**Authors:** Junjie Hang, Binxin Cai, Peng Xue, Lei Wang, Hai Hu, Yangyang Zhou, Shujuan Ren, Jiajin Wu, Meiying Zhu, Donghui Chen, Haiyan Yang, Liwei Wang

**Affiliations:** 1 Department of Oncology and Pancreatic Cancer Center, Shanghai General Hospital, Shanghai Jiao Tong University School of Medicine, Shanghai, China; 2 Shanghai Key Laboratory of Pancreatic Disease, Shanghai, China; 3 Songjiang Center of Shanghai Municipal Center for Disease Control and Prevention, Shanghai, China; University of Arizona, UNITED STATES

## Abstract

**Background:**

Colorectal cancer (CRC) is a major cause of cancer morbidity and mortality. In previous epidemiologic studies, the respective correlation between lifestyle factors and comorbidity and CRC has been extensively studied. However, little is known about their joint effects on CRC.

**Methods:**

We conducted a retrospective case-control study of 1,144 diagnosed CRC patients and 60,549 community controls. A structured questionnaire was administered to the participants about their socio-demographic factors, anthropometric measures, comorbidity history and lifestyle factors. Logistic regression model was used to calculate the odds ratio (ORs) and 95% confidence intervals (95%CIs) for each factor. According to the results from logistic regression model, we further developed healthy lifestyle index (HLI) and comorbidity history index (CHI) to investigate their independent and joint effects on CRC risk.

**Results:**

Four lifestyle factors (including physical activities, sleep, red meat and vegetable consumption) and four types of comorbidity (including diabetes, hyperlipidemia, history of inflammatory bowel disease and polyps) were found to be independently associated with the risk of CRC in multivariant logistic regression model. Intriguingly, their combined pattern- HLI and CHI demonstrated significant correlation with CRC risk independently (OR_HLI_: 3.91, 95%CI: 3.13–4.88; OR_CHI_: 2.49, 95%CI: 2.11–2.93) and jointly (OR: 10.33, 95%CI: 6.59–16.18).

**Conclusions:**

There are synergistic effects of lifestyle factors and comorbidity on the risk of colorectal cancer in the Chinese population.

## Introduction

CRC is a major cause of cancer-related morbidity and mortality. It is the third most commonly diagnosed cancer among males and the second among females on a global level [1]. In China, an annual report on status of cancer in 2011 showed that the incidence and mortality of CRC accounted for 9.20% and 7.09% of all kinds of cancers respectively [2]. Without effective prevention strategy, the male and female CRC incidence rate would reach 33.92/100,000 and 27.13/100,000 in urban areas; and 13.61/100,000 and 13.68/100,000 in rural areas by the year 2015 in China [3]. And such increase in CRC incidence is corresponding with the increasing incidence of CRC-associated comorbidity and adoption of a westernized lifestyle in China. Therefore, understanding the role of these factors may suggest additional prevention strategies that can reduce the incidence of CRC.

Several aspects of risk factors for CRC, including inherited genetic variation [4], lifestyle factors [5,6], comorbidity [7] and the lifetime number of stem cell divisions [8], have been studied on different levels. In this study, we mainly paid our attention to the joint effects of lifestyle factors and comorbidity on CRC risk. In terms of lifestyle factors, there are various known and putative ones including cigarette smoking [9,10], alcohol consumption [11,12], vegetable intake [6], red meat consumption [13], physical activities [14], sleep [15–17] and obesity [18,19]. Meanwhile, several kinds of comorbidity are also linked to an increasing risk of CRC such as chronic gastritis [20–22], schistosomiasis [23], inflammatory bowel disease (IBD)[24], polyps [25], diabetes [26,27], hyperglycemia and hypertension [28,29].

All these evidence suggests the multi-factorial nature and preventive potential of CRCs with optimal lifestyle and well-controlled comorbidity. However, to our knowledge, no study has been done to determine the joint effects of lifestyle factors and comorbidity on colorectal carcinogenesis, especially in China. Given this condition, we embark on the present case-control study with a view to assess the potential influence of several factors on CRC in Chinese population more precisely. And we established Healthy Lifestyle Index (HLI) and Comorbidity History Index (CHI) to evaluate possible synergistic effects of lifestyle factors and comorbidity on the risk for CRC.

## Materials and Methods

### Study population

Colorectal cancer cases were drawn from a database of the Songjiang Center of Shanghai Municipal Center for Disease Control and Prevention in China. In brief, cases were patients who had a histopathologic diagnosis of primary invasive colorectal cancer (ICD, Tenth Revision, codes C18–C20). The exclusion criteria were presence of unknown primary tumors, concurrent cancer at another organ site, past history of cancer and incomplete records. From January 2001 to December 2012, a total of 1,144 cases were eligible for analysis.

Controls were randomly selected from local residents and considered cancer-free at the time of enrollment. In total, 60,549 controls were included in this study after excluding 3,167 (4.9%) people with incomplete information. Males accounted for 45.2% of the whole group. Among these people, the following inclusion criteria were applied: Shanghai residency, ability to communicate in Shanghai dialect or mandarin and maintain the mental and physical ability to participate in an interview about 0.5 hour with structured questionnaires. Written informed consent was obtained from each participant. Ethical approval was obtained from the Ethics Committee of Shanghai General Hospital.

### Data collection

Baseline characteristics of the study population were assessed by means of interviews with structured questionnaires. The content of the questionnaire focused on socio-demographic variables, anthropometric measures, comorbidity history, family history of colorectal cancer and lifestyle factors. The demographic data was composed of age, gender and educational level. Anthropometric measurements included height and weight. Body mass index (BMI) was further calculated from height and weight. And comorbidity history was comprised of the history of IBD, colorectal polyps, gastritis, schistosomiasis, diabetes, hypertension and hyperlipidemia. In addition, lifestyle factors included smoking, alcohol consumption, sleep, physical activities, red meat and vegetable consumption.

All lifestyle factors were collected by self-report. Inquiry on smoking history included three status (never, former or current). Alcohol use was also divided into three categories (never, former or current) and the latter two groups were asked to choose from three types of alcoholic beverages (beer, wine/yellow rice wine, and whiskey/Chinese hard liquor). Physical activities were assessed by the following question-'On average, how many times in a week did you exercise (a time means a minimum of 1 hour moderate-intensity or 30 min vigorous-intensity exercise [30])?', with response categories of never or hardly, 1–2 times/week, 3–5 times/week, 6–7 times/week. Vegetable consumption was assessed using four categories: <100 g/day, <300 g/day, <500 g/day and ≥500 g/day [31]. Red meat was considered the intake of mutton, lamb, veal, pork, and beef [32] and was assessed using the following question-'On average, how many days in a week did you eat meat?', with response categories of never or hardly, 1–2 days/week, 3–4 days/week, 5–7 days/week. We further categorized duration of sleep into five magnitudes (<6, 6, 7, 8, ≥ 9 hours/day)[16].

### Healthy lifestyle index (HLI) and comorbidities history index (CHI)

With reference to the definition of Healthy Lifestyle Index (HLI) in a large European cohort study [33], we investigated the association between combined lifestyle factors and the risk for CRC. The HLI presented in S1 Table was modified on the basis of knowledge of CRC risks, the published data and the results from multivariate logistic regression model. The HLI score was derived by assigning a score of 1 respectively to the following behaviors: exercise 3 or more times per week [34], sleep duration of less than 8 hours/day [17] and having a healthy diet which means limited red meat consumption and ample servings of vegetables [6,32]. Then we calculated the HLI scores by summing the individual scores for each of the four-lifestyle factors. We further developed the Comorbidity History Index (CHI) in a similar way (S1 Table).

### Statistical analysis

All statistical analyses were performed with SPSS statistical software (version 21.0, SPSS Inc, Chicago, IL, USA). First, baseline characteristics (including sex, age, BMI, socio-demographic characteristics, lifestyle factors, family history, and comorbidity history) of the population in study were presented. To make the model convenient enough to be applied in practice, we turned polytomous variables into binary categorical variables. Differences in lifestyle factors and comorbidity history between cases and controls were assessed with Pearson’s chi-square test. Two-sided P<0.05 was considered statistically significant. Then, logistic regression model was used to estimate the effects of these factors on risk for CRC. For each factor, we calculated the ORs and corresponding 95%CIs. All ORs were adjusted for age, sex, BMI, education level and family history of CRC. P-values in all models were calculated using Wald tests.

Factors that remained significantly correlated with the risk of CRC in logistic regression model were selected to generate the Healthy Lifestyle Index (HLI) score and Comorbidity History Index (CHI) score for evaluation of the combined effects on the risk of CRC. And we further stratified HLI and CHI scores into three categories, respectively, with the cut-off points made based on the distribution of the subjects (S4 Table). Our goal was to have as many as possible samples in each category to provide stability to estimate risk. Logistic regression model was used to estimate the covariates (including sex, age, BMI, educational level and history of CRC in first-degree relatives) adjusted ORs and corresponding 95%CIs for the association between lifestyle factors, comorbidity history and CRC risk.

Lastly, we accessed the joint effects of lifestyle factors and comorbidity history on CRC risk with additive models [35]. Because HLI and CHI scores both had three categories, by crossing them, we had a new variable which included nine possible combinations, as was shown in S5 Table. The new variable was named HLI&CHI and assigned values of numbers from 1 to 9, which was in accordance with the nine categories. And the last of these categories, the combination of high HLI score and high CHI score, served as the reference category in the logistic regression models. ORs and 95%CIs were estimated from logistic regression models adjusted for sex, age, BMI, educational level and history of CRC in first-degree relatives. Furthermore, to evaluate deviation from the additive model, we calculated the synergy index (S = [OR_HLI&CHI_-1]/[OR_HLI_+OR_CHI_-2])[36]. If there is no biological interaction, S is equal to 1. Accordingly, S>1 means synergy interactions while S<1 means antagonistic interactions between lifestyle factors and comorbidity history [37].

## Results

Of the 1,144 subjects, 52.9% were males while 45.2% were males in 60,549 controls. The median ages of cases and controls were 62 (range: 24 to 97) and 69(range: 23 to 98), respectively. The distributions of socio-demographic, lifestyle, and comorbidity characteristics of cases and controls were presented in S2 Table. Compared with controls, a greater proportion of cases have lower educational level, history of CRC in first-degree relatives and normal BMI (18.0–25.0 kg/m^2^). During subsequent statistical analyses, we adjusted for all demographic factors in unconditional logistic regression models.

S3 Table shows the results of Pearson’s chi-square test and the correlation of lifestyle factors and comorbidity with CRC risk. High and very high frequency of physical activities, shorter sleep duration and healthy vegetable consumption were found to be correlated with a reduced risk. On the contrast, elevated risks of CRC were observed in association with red meat consumption, diabetes, hyperlipidemia and history of IBD and polyps. Intriguingly, we observed that smoking status, alcohol consumption, hypertension, schistosomiasis and gastritis were not correlated with the risk of CRC independently.

We further developed HLI through a composite score of those lifestyle variables (including physical activity, shorter sleep duration, red meat and vegetable consumption) that independently influenced CRC risk. Similarly, we created CHI that summarized individual risk across comorbidity (including diabetes, hyperlipidemia and history of IBD or polyps) that was associated individually with CRC risk. We stratified both HLI and CHI scores into three categories (S4 Table). The low HLI score was related to an approximately 4-fold elevated risk of overall CRC when compared with high HLI score. And the median HLI score exhibited a 1.97-fold increase in the risk. In addition, low and median CHI scores also resulted in 2.49-fold and 1.74-fold elevated risk when compared to high CHI score.

The magnitude of the combined risk was demonstrated in S5 Table. Notably, a 10.33-fold (95%CI: 6.59–16.18) of increase in risk of CRC was exhibited when HLI and CHI scores were low. While healthy lifestyle, demonstrated as high HLI score, could modify risk of CRC at a median level of comorbidity risk with an estimated synergistic index (S) equal to 0.92, it lost protective benefits when a high level of comorbidity risk existed (S = 1.03). Likewise, good general physical condition without any comorbidity was able to in part offset the risk of CRC when HLI score was median (S = 0.89) but lost such effect when HLI score was low (S = 1.02). In addition, when HLI and CHI were both at low/median level, their combined risk would exceed the sum of the risks for each of them alone (S from 1.36 to 2.12).

## Discussion

**Fig 1:**
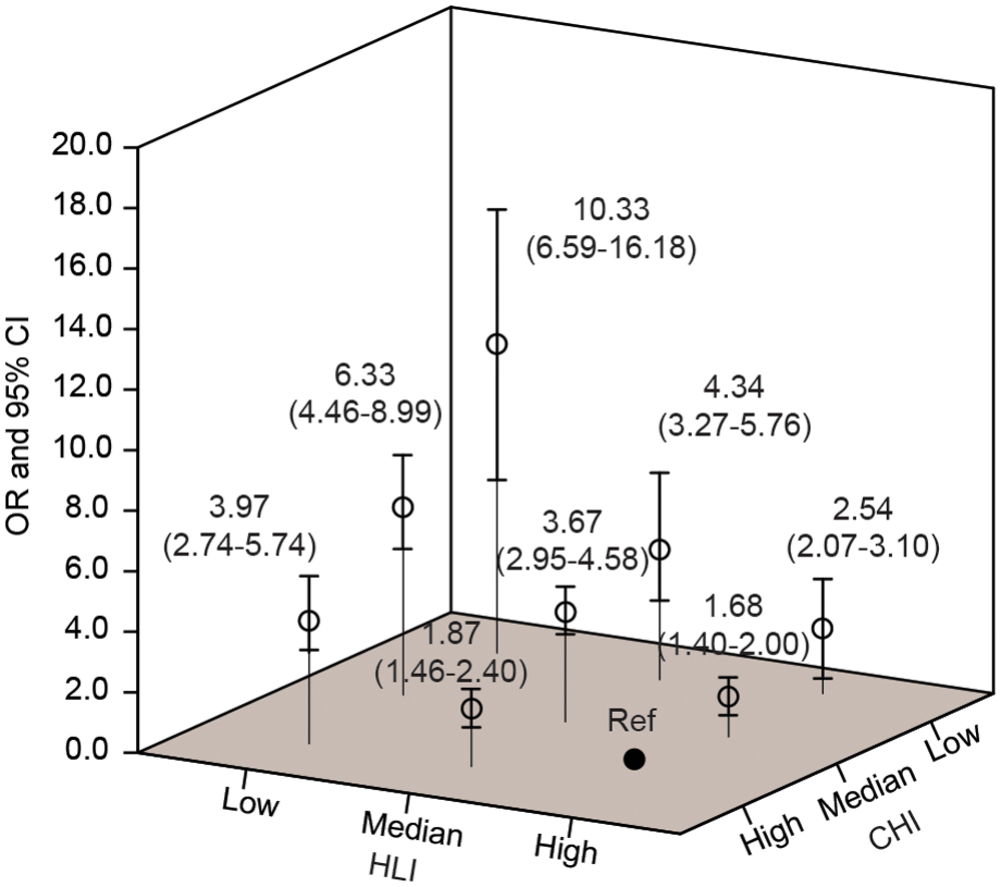
The joint effects of lifestyle factors and comorbidities history on colorectal cancer risk. Error bars indicate the 95%CI.

In this present population-based case-control study, we had three major findings: (1) we confirmed the results of published studies that four lifestyle factors (including physical activities, sleep time, red meat and vegetable consumption) and four types of comorbidity (including diabetes, hyperlipidemia, IBD and polyps) were independently correlated with CRC risk; (2) we observed no significant correlation between some universally-recognized risk factors (like alcohol consumption) and CRC; (3) we further demonstrated the joint effects of lifestyle factors and comorbidity on the risk for CRC (Fig 1). And to our knowledge, this is the first study to evaluate such combined impact on CRC risk.

Multiple published studies linked lifestyle factors individually to the risk of CRC, whereas only few of them examined the combined effects of these factors [33, 38–40]. In a large European cohort study published in 2014, a combination of five healthy lifestyle behaviors—healthy weight, physical activities, non-smoking, limited alcohol consumption and a healthy diet contributed to a lower incidence of CRC [33]. In 2013, Andrew et, al showed that a designed protective lifestyle factor index was associated with reduced age- and sex-standardized incidence rates of CRC [39]. Likewise, a couple of other studies [38, 40] also came out with similar results with their unique inclusion of factors.

In our study, we evaluated six lifestyle factors at the beginning and excluded smoking as well as alcohol consumption from HLI score according to the results of multivariate analysis. Although it is universally acknowledged that alcohol consumption plays an important role in the colorectal oncogenesis [11] by various mechanisms including inhibiting the expression of the p16 gene [12], few studies have taken the type and ingredients of alcoholic beverages into consideration. To our knowledge, there are discrepancies regarding the specific effects of different types of alcoholic beverages on the cancer, and such effects are mainly due to their alcoholic content (ethanol) or their non-alcoholic components [41]. For instance, red wine polyphenols, which is consisted of various powerful antioxidants like resveratrol, have been studied extensively for their chemo-preventive activity in cancer [42]. In our study, 10,096 of 19,316 alcohol users mainly consumed red wine or yellow rice wine, which may partially explain why alcohol consumption is not independently associated with CRC risk in this study. Besides, smoking and alcohol consumption, both taken as longitudinal behavior, might change after the diagnosis in those CRC participants. And such reverse causation would lead to the results that some CRC cases shifted from the current group to the former group, which was in accordance with our data- the current group was significantly smaller in cases than controls but the former group was larger. Given this situation, when we turned polytomous variables into binary categorical variables, we took current and former smokers (drinkers) as a group while never-smokers (never-drinkers) as the other group. And we hope such stratification can minimize the effects from reverse causation.

In addition, most of published studies laid emphasis on the effect of a single kind of comorbidity, such as IBD, while few of them mainly focused on the relationship between the risk of CRC and metabolism syndrome, which took hypertension, diabetes and hyperlipidemia into consideration simultaneously. It is universally acknowledged that the history of IBD, colorectal polyps and diabetes can be attributed to an increased risk of developing CRC. For example, the imbalance between pro- and anti-inflammatory cytokines (IL-6, TNF-α and so on), oxidative DNA damage and genomic instability have all been implicated in the process of colorectal carcinogenesis in IBD patients [24]. On the contrast, the correlation between the history of gastritis, schistosomiasis, hypertension, hyperlipidemia and CRC risk is mainly observed in epidemiological studies [21,23,28].

The seven comorbidity mentioned before were included in our study initially. We further created CHI with the exclusion of the history of gastritis, schistosomiasis and hypertension because these factors were not independently associated with CRC risk in multivariate analysis. Such result basically corresponded with other published data. For example, hypertension has been demonstrated to increase the risk of CRC separately as an individual component of metabolic syndrome, although the correlation was not significant and the mechanism was far from clear [28].

Our composite score model offers a tool not only to estimate how lifestyle factors and comorbidity affect the risk of CRC individually but also manifests the synergistic effects of them. And the establishment of such model is based on the results of Tomas Anderson's work in assessment of biological interaction [37] in 2005. When HLI and CHI are both at low/median level, their combined risk will exceed the sum of the risks for each of them alone, which indicates that people belonging to this group require targeted support and services. And this emerging intelligence can be used by some national institutions to optimize the early screening program for CRC. Another highlight of this study is the finding that healthy lifestyle can modify risk of CRC at a median level of comorbidity risk but loses protective benefits when a high level of comorbidity risk existed, which suggests the former group should especially perceive themselves to be knowledgeable about the importance in changing those at-risk lifestyle habits.

There are some strengths of this study present here. First, our study boasts a large sample size of 1,144 CRC cases and 60,549 controls. Second, most variables included in this model are commonly available from the patients' history which ensures the results of the model to be validated easily in other studies in the future. We also acknowledge several limitations. First, questionnaire-based description of lifestyle factors and comorbidity history are subject to error and may reflect the responders' belief that particular choices of answers are desirable. Second, some suspected risk factors for CRC are not included in our study, such as the use of aspirin. Third, the questionnaire might not be precise enough to answer the degree or strength of associations with some factors. For example, when it comes to sleep, we only take sleep duration into the questionnaire, but some studies also showed sleep quality, which included insomnia and snoring, could also affect the risk of CRC [16]. However, the complexities of questionnaire maybe affect the completion of it because many aged participants don't have such patience and mental ability to complete it. In addition, we mainly focused on patterns of lifestyle and comorbidities rather than specific individual exposures in this study. Finally, although we set the cut-off points for risk factors with reference to the results from studies published before, the estimates of correlation would unavoidably vary depending on where cut-off points were set.

## Conclusions

There are synergistic effects of lifestyle factors and comorbidity on the risk of colorectal cancer in Chinese population characterized by a gradually westernized lifestyle. And these results could have important public health implications-both healthy lifestyles and well-controlled comorbidity may lead to a longer, healthier and cancer-free life. In addition, we anticipate future work using data of genomic assessments, molecular markers and the lifetime number of stem cell divisions in colon to further demonstrate the comprehensive effects of all these factors on the risk of CRC with a more precise model.

## Supporting Information

S1 Table Description of factors comprising Healthy Lifestyle Index (HLI) and Comorbidities History Index (CHI). Abbreviation: IBD, inflammatory bowel disease.
(DOCX)

S2 Table Baseline characteristics of the study population in the large Chinese case-control study. Abbreviation: BMI, body mass index; CRC, colorectal cancer; IBD, inflammatory bowel disease.
(DOCX)

S3 Table Lifestyle factors, comorbidities history and their independent association with risk of CRC. P_1_: P values of variables in Pearson’s chi-square test; P_2_: P values of variables in multivariate logistic regression model which are adjusted for all variables in the S2 Table with age and BMI as continuous variables.
(DOCX)

S4 Table Odds ratios of CRC in relation to HLI and CHI score individually. Abbreviation: HLI, Healthy Lifestyle Index; CHI, Comorbidities History Index. OR was adjusted for sex, age, BMI, educational level and history of colorectal cancer in first-degree relatives.
(DOCX)

S5 Table Synergistic effects of lifestyle factors and comorbidities history on colorectal cancer risk. Abbreviation: S, the synergism index; OR was adjusted for sex, age, BMI, educational level and history of colorectal cancer in first-degree relatives.
(DOCX)
