# Supplementary material for: The Joint Effects of Lifestyle Factors and Comorbidities on the Risk of Colorectal Cancer: A Large Chinese Retrospective Case-Control Study
Source: PLoS One. 2015 Dec 28;10(12):e0143696. doi: 10.1371/journal.pone.0143696 (PMC4692389; doi:10.1371/journal.pone.0143696)
Supplement: S1 Table — Abbreviation: IBD, inflammatory bowel disease. (DOCX) [file pone.0143696.s001.docx]

**Table 1.Description of factors comprising** **Healthy Lifestyle Index (HLI) and Comorbidities History Index (CHI)**

| **Variables** | **Index** | **Description** |
| --- | --- | --- |
| Physical activity | 0 | Low physical activity: exercise out <3 times/week |
|  | 1 | High physical activity: exercise out >3 times/week |
| Sleep duration | 0 | Longer sleep duration: >8h/d |
|  | 1 | Normal sleep duration: <8h/d |
| Red meat consumption | 0 | Unhealthy consumption quality: consuming red meat >3 days/week |
|  | 1 | Healthy consumption quality: consuming red meat <3 days/week |
| Vegetable consumption | 0 | Unhealthy consumption quality:<300 g/day |
|  | 1 | Healthy consumption quality: >300 g/day |
| Diabetes | 0 | Yes |
|  | 1 | No |
| Hyperlipidemia | 0 | Yes |
|  | 1 | No |
| History of IBD | 0 | Yes |
|  | 1 | No |
| History of polyps | 0 | Yes |
|  | 1 | No |

Abbreviation: IBD, inflammatory bowel disease.
