# Supplementary material for: The Joint Effects of Lifestyle Factors and Comorbidities on the Risk of Colorectal Cancer: A Large Chinese Retrospective Case-Control Study
Source: PLoS One. 2015 Dec 28;10(12):e0143696. doi: 10.1371/journal.pone.0143696 (PMC4692389; doi:10.1371/journal.pone.0143696)
Supplement: S2 Table — Abbreviation: BMI, body mass index; CRC, colorectal cancer; IBD, inflammatory bowel disease. (DOCX) [file pone.0143696.s002.docx]

**Table 2.Baseline characteristics of the study population in the large Chinese case-control study**

|  | **Control** | | **Case** | |
| --- | --- | --- | --- | --- |
| **characteristics** | **N** | **%** | **N** | **%** |
| Sex |  |  |  |  |
| male | 27,345 | 45.2 | 605 | 52.9 |
| female | 33,204 | 54.8 | 539 | 47.1 |
| Age |  |  |  |  |
| <60 years | 22,099 | 36.5 | 211 | 18.4 |
| 60-69 years | 27,714 | 45.8 | 362 | 31.6 |
| >70 years | 10,736 | 17.7 | 571 | 49.9 |
| BMI |  |  |  |  |
| Underweight(<18.0) | 1,704 | 2.8 | 96 | 8.4 |
| Normal weight(18.0–25.0) | 34,803 | 57.5 | 688 | 60.1 |
| Overweight(>25.0) | 24,042 | 39.7 | 360 | 31.5 |
| Educational level |  |  |  |  |
| illiteracy | 12,307 | 20.3 | 282 | 24.7 |
| low | 24,467 | 40.4 | 451 | 39.4 |
| intermediate | 22,147 | 36.6 | 347 | 30.3 |
| high | 1,628 | 2.7 | 64 | 5.6 |
| Family history of CRC |  |  |  |  |
| Yes | 2,033 | 3.4 | 97 | 8.5 |
| No | 58,516 | 96.6 | 1,047 | 91.5 |
| Smoking status |  |  |  |  |
| Never smoker | 41,620 | 68.7 | 757 | 66.2 |
| Former smoker | 2,955 | 4.9 | 242 | 21.2 |
| Current smoker | 15,974 | 26.4 | 145 | 12.7 |
| Alcohol consumption |  |  |  |  |
| Never | 49,311 | 81.4 | 936 | 81.8 |
| Former | 783 | 1.3 | 72 | 6.3 |
| Current | 10,455 | 17.3 | 136 | 11.9 |
| Physical activity |  |  |  |  |
| Never or hardly | 11,345 | 18.7 | 301 | 26.3 |
| 1-2 time/week | 8,357 | 13.8 | 193 | 16.9 |
| 3-5 time/week | 12,884 | 21.3 | 312 | 27.3 |
| 6-7 time/week | 27,963 | 46.2 | 338 | 29.5 |
| Sleep |  |  |  |  |
| <6 hours/day | 6,799 | 11.2 | 100 | 8.7 |
| 6 hours/day | 13,658 | 22.6 | 195 | 17 |
| 7 hours/day | 16,307 | 26.9 | 256 | 22.4 |
| 8 hours/day | 20,427 | 33.7 | 388 | 33.9 |
| >9 hours/day | 3,358 | 5.5 | 205 | 17.9 |
| Red meat consumption |  |  |  |  |
| Never or hardly | 3,370 | 5.6 | 64 | 5.6 |
| 1-2 days/week | 9,631 | 15.9 | 153 | 13.4 |
| 3-4 days/week | 25,624 | 42.3 | 413 | 36.1 |
| 5-7 days/week | 21,924 | 36.2 | 514 | 44.9 |
| Vegetable consumption |  |  |  |  |
| <100 g/day | 1,339 | 2.2 | 33 | 2.9 |
| <300 g/day | 7,174 | 11.8 | 217 | 19 |
| <500 g/day | 32,477 | 53.6 | 563 | 49.2 |
| >500 g/day | 19,559 | 32.3 | 331 | 28.9 |
| Diabetes |  |  |  |  |
| Yes | 5,341 | 8.8 | 137 | 12 |
| No | 55,208 | 91.2 | 1,007 | 88 |
| Hypertension |  |  |  |  |
| Yes | 21,219 | 35 | 468 | 40.9 |
| No | 39,330 | 65 | 676 | 59.1 |
| Hyperlipidemia |  |  |  |  |
| Yes | 3,137 | 5.2 | 92 | 8 |
| No | 57,412 | 94.8 | 1,052 | 92 |
| History of IBD |  |  |  |  |
| Yes | 2,534 | 4.2 | 155 | 13.5 |
| No | 58,015 | 95.8 | 989 | 86.5 |
| History of polyps |  |  |  |  |
| Yes | 1,731 | 2.9 | 154 | 13.5 |
| No | 58,818 | 97.1 | 990 | 86.5 |
| Schistosomiasis |  |  |  |  |
| Yes | 9,204 | 15.2 | 245 | 21.4 |
| No | 51,345 | 84.8 | 899 | 78.6 |
| Gastritis |  |  |  |  |
| Yes | 8,595 | 14.2 | 193 | 16.9 |
| No | 51,954 | 85.8 | 951 | 83.1 |

Abbreviation: BMI, body mass index; CRC, colorectal cancer; IBD, inflammatory bowel disease.
