# Supplementary material for: The Joint Effects of Lifestyle Factors and Comorbidities on the Risk of Colorectal Cancer: A Large Chinese Retrospective Case-Control Study
Source: PLoS One. 2015 Dec 28;10(12):e0143696. doi: 10.1371/journal.pone.0143696 (PMC4692389; doi:10.1371/journal.pone.0143696)
Supplement: S3 Table — P1: P values of variables in Pearson’s chi-square test; P2: P values of variables in multivariate logistic regression model which are adjusted for all variables in the S2 Table with age and BMI as continuous variables. (DOCX) [file pone.0143696.s003.docx]

**Table 3.Lifestyle factors, comorbidities history and their independent association with risk of CRC**

| **Characteristics** | **Control** | **Case** | **P_1_** | **OR** | **95%CI** | **P_2_** |
| --- | --- | --- | --- | --- | --- | --- |
| Smoking status |  |  |  |  |  |  |
| Never smoker | 41,620 | 757 | 0.06 | 1.02 | 0.86-1.22 | 0.82 |
| Former/current smoker | 18,929 | 387 |  | 1 |  |  |
| Alcohol consumption |  |  |  |  |  |  |
| Never | 49,311 | 936 | 0.74 | 1.08 | 0.91-1.29 | 0.38 |
| Former/current | 11,238 | 208 |  | 1 |  |  |
| Physical activity |  |  |  |  |  |  |
| ≥3 times/week | 40,847 | 650 | <0.001 | 0.64 | 0.56-0.72 | <0.001 |
| <3 times/week | 19,702 | 494 |  | 1 |  |  |
| Sleep |  |  |  |  |  |  |
| <8 hours/day | 36,764 | 551 | <0.001 | 0.56 | 0.50-0.63 | <0.001 |
| ≥8 hours/day | 23,785 | 593 |  | 1 |  |  |
| Red meat consumption |  |  |  |  |  |  |
| <3 days/week | 13,001 | 927 | 0.04 | 0.83 | 0.71-0.97 | 0.02 |
| >3 days/week | 47,548 | 217 |  | 1 |  |  |
| Vegetable consumption |  |  |  |  |  |  |
| >300 g/day | 52,036 | 894 | <0.001 | 0.69 | 0.59-0.80 | <0.001 |
| <300 g/day | 8,513 | 250 |  | 1 |  |  |
| Diabetes |  |  |  |  |  |  |
| Yes | 5,341 | 137 | <0.001 | 1.24 | 1.02-1.50 | 0.03 |
| No | 55,208 | 1,007 |  | 1 |  |  |
| Hypertension |  |  |  |  |  |  |
| Yes | 21,219 | 468 | <0.001 | 0.91 | 0.80-1.04 | 0.17 |
| No | 39,330 | 676 |  | 1 |  |  |
| Hyperlipidemia |  |  |  |  |  |  |
| Yes | 3,137 | 92 | <0.001 | 1.33 | 1.05-1.68 | 0.02 |
| No | 57,412 | 1,052 |  | 1 |  |  |
| History of IBD |  |  |  |  |  |  |
| Yes | 2,534 | 155 | <0.001 | 2.52 | 2.07-3.06 | <0.001 |
| No | 58,015 | 989 |  | 1 |  |  |
| History of polyps |  |  |  |  |  |  |
| Yes | 1,731 | 154 | <0.001 | 3.72 | 3.07-4.50 | <0.001 |
| No | 58,818 | 990 |  | 1 |  |  |
| Schistosomiasis |  |  |  |  |  |  |
| Yes | 9,204 | 245 | <0.001 | 1.05 | 0.90-1.22 | 0.54 |
| No | 51,345 | 899 |  | 1 |  |  |
| Gastritis |  |  |  |  |  |  |
| Yes | 8,595 | 193 | 0.01 | 0.88 | 0.75-1.05 | 0.16 |
| No | 51,954 | 951 |  | 1 |  |  |

P_1_: P values of variables in Pearson’s chi-square test; P_2_: P values of variables in multivariate logistic regression model which are adjusted for all variables in the S2 Table with age and BMI as continuous variables.
