# Supplementary material for: The Joint Effects of Lifestyle Factors and Comorbidities on the Risk of Colorectal Cancer: A Large Chinese Retrospective Case-Control Study
Source: PLoS One. 2015 Dec 28;10(12):e0143696. doi: 10.1371/journal.pone.0143696 (PMC4692389; doi:10.1371/journal.pone.0143696)
Supplement: S4 Table — Abbreviation: HLI, Healthy Lifestyle Index; CHI, Comorbidities History Index. OR was adjusted for sex, age, BMI, educational level and history of colorectal cancer in first-degree relatives. (DOCX) [file pone.0143696.s004.docx]

**Table 4.Odds ratios of CRC in relation to HLI and CHI score individually**

|  | **Control** | | **Case** | |  |  |  |
| --- | --- | --- | --- | --- | --- | --- | --- |
| **Index** | **n** | **%** | **n** | **%** | **OR** | **95%CI** | **P** |
| HLI |  |  |  |  |  |  |  |
| low(0-1) | 9,218 | 15.2 | 298 | 26 | 3.91 | 3.13-4.88 | <0.001 |
| med(2) | 24,156 | 39.9 | 509 | 44.5 | 1.97 | 1.71-2.26 | <0.001 |
| high(3-4) | 27,175 | 44.9 | 337 | 29.5 | 1 |  |  |
| CHI |  |  |  |  |  |  |  |
| low(0-2) | 1,535 | 2.5 | 108 | 9.4 | 2.49 | 2.11-2.93 | <0.001 |
| med(3) | 9,545 | 15.8 | 307 | 26.8 | 1.74 | 1.51-2.01 | <0.001 |
| high(4) | 49,469 | 81.7 | 729 | 63.7 | 1 |  |  |

Abbreviation: HLI, Healthy Lifestyle Index; CHI, Comorbidities History Index.

OR was adjusted for sex, age, BMI, educational level and history of colorectal cancer in first-degree relatives.
