# Supplementary material for: The Joint Effects of Lifestyle Factors and Comorbidities on the Risk of Colorectal Cancer: A Large Chinese Retrospective Case-Control Study
Source: PLoS One. 2015 Dec 28;10(12):e0143696. doi: 10.1371/journal.pone.0143696 (PMC4692389; doi:10.1371/journal.pone.0143696)
Supplement: S5 Table — Abbreviation: S, the synergism index; OR was adjusted for sex, age, BMI, educational level and history of colorectal cancer in first-degree relatives. (DOCX) [file pone.0143696.s005.docx]

**Table 5.Synergistic effects of lifestyle factors and comorbidities history on colorectal cancer risk**

| **HLI** | **CHI** | **HLI&CHI** | **Control (%)** | **Case (%)** | **OR** | **95%CI** | **P** | **S** |
| --- | --- | --- | --- | --- | --- | --- | --- | --- |
| low | low | 1 | 224(0.4) | 27(2.4) | 10.33 | 6.59-16.18 | <0.001 | 2.12 |
| low | med | 2 | 560(0.9) | 44(3.8) | 6.33 | 4.46-8.99 | <0.001 | 1.46 |
| low | high | 3 | 751(1.2) | 37(3.2) | 3.97 | 2.74-5.74 | <0.001 | 1.02 |
| med | low | 4 | 1,393(2.3) | 72(6.3) | 4.34 | 3.27-5.76 | <0.001 | 1.36 |
| med | med | 5 | 3,627(6.0) | 143(12.5) | 3.67 | 2.95-4.58 | <0.001 | 1.56 |
| med | high | 6 | 4,525(7.5) | 92(8.0) | 1.87 | 1.46-2.40 | <0.001 | 0.89 |
| high | low | 7 | 7,601(12.6) | 199(17.4) | 2.54 | 2.07-3.10 | <0.001 | 1.03 |
| high | med | 8 | 19,969(33.0) | 322(28.1) | 1.68 | 1.40-2.00 | <0.001 | 0.92 |
| high | high | 9 | 21,899(36.2) | 208(18.2) | 1 |  |  |  |

Abbreviation: S, the synergism index;

OR was adjusted for sex, age, BMI, educational level and history of colorectal cancer in first-degree relatives.
